# Differential effects of the poly (ADP-ribose) polymerase (PARP) inhibitor NU1025 on topoisomerase I and II inhibitor cytotoxicity in L1210 cells in vitro

**DOI:** 10.1054/bjoc.2000.1555

**Published:** 2001-01-01

**Authors:** K J Bowman, D R Newell, A H Calvert, N J Curtin

**Affiliations:** Cancer Research Unit, University of Newcastle upon Tyne Medical School, Framlington Place, Newcastle upon Tyne, NE2 4HH, UK

**Keywords:** poly(ADP-ribose) polymerase, topoisomerase I, topoisomerase II, cytotoxicity, DNA damage

## Abstract

The potent novel poly(ADP-ribose) polymerase (PARP) inhibitor, NU1025, enhances the cytotoxicity of DNA-methylating agents and ionizing radiation by inhibiting DNA repair. We report here an investigation of the role of PARP in the cellular responses to inhibitors of topoisomerase I and II using NU1025. The cytotoxicity of the topoisomerase I inhibitor, camptothecin, was increased 2.6-fold in L1210 cells by co-incubation with NU1025. Camptothecin-induced DNA strand breaks were also increased 2.5-fold by NU1025 and exposure to camptothecin-activated PARP. In contrast, NU1025 did not increase the DNA strand breakage or cytotoxicity caused by the topoisomerase II inhibitor etoposide. Exposure to etoposide did not activate PARP even at concentrations that caused significant levels of apoptosis. Taken together, these data suggest that potentiation of camptothecin cytotoxicity by NU1025 is a direct result of increased DNA strand breakage, and that activation of PARP by camptothecin-induced DNA damage contributes to its repair and consequently cell survival. However, in L1210 cells at least, it would appear that PARP is not involved in the cellular response to etoposide-mediated DNA damage. On the basis of these data, PARP inhibitors may be potentially useful in combination with topoisomerase I inhibitor anticancer chemotherapy. © 2001 Cancer Research Campaign http://www.bjcancer.com


*British Journal of Cancer* (2001) **84**(1), 106–112
© 2001 Cancer Research Campaign
doi: 10.1054/ bjoc.2000.1555, available online at http://www.idealibrary.com on IDEAL®

http://www.bjcancer.com

### Differential effects of the poly (ADP-ribose) polymerase (PARP) inhibitor NU1025 on topoisomerase I and II inhibitor cytotoxicity in L1210 cells in vitro

**KJ Bowman*, DR Newell, AH Calvert and NJ Curtin**

Cancer Research Unit, University of Newcastle upon Tyne Medical School, Framlington Place, Newcastle upon Tyne NE2 4HH, UK

**Summary** The potent novel poly(ADP-ribose) polymerase (PARP) inhibitor, NU1025, enhances the cytotoxicity of DNA-methylating agents and ionizing radiation by inhibiting DNA repair. We report here an investigation of the role of PARP in the cellular responses to inhibitors of topoisomerase I and II using NU1025. The cytotoxicity of the topoisomerase I inhibitor, camptothecin, was increased 2.6-fold in L1210 cells by co-incubation with NU1025. Camptothecin-induced DNA strand breaks were also increased 2.5-fold by NU1025 and exposure to camptothecin-activated PARP. In contrast, NU1025 did not increase the DNA strand breakage or cytotoxicity caused by the topoisomerase II inhibitor etoposide. Exposure to etoposide did not activate PARP even at concentrations that caused significant levels of apoptosis. Taken together, these data suggest that potentiation of camptothecin cytotoxicity by NU1025 is a direct result of increased DNA strand breakage, and that activation of PARP by camptothecin-induced DNA damage contributes to its repair and consequently cell survival. However, in L1210 cells at least, it would appear that PARP is not involved in the cellular response to etoposide-mediated DNA damage. On the basis of these data, PARP inhibitors may be potentially useful in combination with topoisomerase I inhibitor anticancer chemotherapy. © 2001 Cancer Research Campaign http://www.bjcancer.com

**Keywords**: poly(ADP-ribose) polymerase; topoisomerase I; topoisomerase II; cytotoxicity; DNA damage

The nuclear enzyme, poly(ADP-ribose) polymerase (PARP), is activated in response to DNA strand breaks and is strongly implicated in the repair of such lesions (reviewed in de Murcia and Menissier-de Murcia 1994). It has therefore been proposed that inhibition of PARP may increase the efficacy of DNA-damaging anticancer therapy. Indeed, inhibition of PARP has been demonstrated to increase the cytotoxicity of several anticancer agents (reviewed in Griffin et al, 1995*a*). However, in comparison to the well-defined role of PARP in the repair of methylating agent- and γ-irradiation-induced DNA damage, the role of PARP in response to topoisomerase inhibitor-mediated DNA damage has not been extensively studied.

Topoisomerases, which catalyse the DNA breakage, unwinding and religation necessary to relieve torsional strain, are the molecular target of many anticancer agents. In particular, the observation that topoisomerase I is elevated in some tumours (Kaufmann et al, 1995), has led to increased interest in the use of topoisomerase I inhibitors in the treatment of cancer. Evidence concerning the role of PARP in response to treatment with topoisomerase inhibitors is scant and contradictory. For example, the DNA intercalating topoisomerase II inhibitor, doxorubicin, stimulated PARP activity in L1210 cells in one study (Daugherty et al, 1988) but not in another (Zwelling et al, 1982). DNA strand breaks, and hence cytotoxicity, produced by topoisomerase inhibitors correlates directly with topoisomerase activity, and in isolated enzyme studies PARP polyADP-ribosylates topoisomerases I and II, down-regulating their activity (Ferro and Olivera, 1984; Darby et al, 1985). Consistent with these observations Mattern et al (1987) demonstrated potentiation of camptothecin and teniposide cytotoxicity by the PARP inhibitor, 3-aminobenzamide (3AB), in L1210 cells. They suggested that topoisomerase I and II activity is normally down-regulated by polyADP-ribosylation, hence sensitization to camptothecin and teniposide caused by inhibition of PARP was due to de-repression of topoisomerase activity rather than a direct effect of PARP inhibition on DNA repair. However, subsequent studies in CCRF CEM cells failed to demonstrate potentiation of etoposide by 3AB (Marks and Fox, 1991).

As a PARP inhibitor, 3AB lacks potency and specificity (Milam et al, 1986; Eriksson et al, 1996). It has been shown that potentiation of the activity of cytotoxic drugs by 3AB can be via PARP-independent mechanisms (Moses et al, 1988*a*; 1988*b*; 1990). Recently, more potent PARP inhibitors have been identified (Suto et al, 1991; Banasik et al, 1992) and these have been used to clarify more precisely the role of PARP as a determinant of the activity of cytotoxic drugs, for example cisplatin (Bernges and Zeller, 1996). NU1025 (8-hydroxy-2-methyl-quinazolin-4-[3*H*]one), which is approximately 50 × more potent than 3AB as a PARP inhibitor (Griffin et al, 1995*b*; 1996) has recently been evaluated as a resistance modifier. NU1025 can enhance the cytotoxicity of some classes of DNA-damaging agents (monofunctional DNA-alkylating agents, γ-irradiation and bleomycin) but not others (antimetabolites) in L1210 cells (Bowman et al, 1998). In order to investigate the potential of PARP inhibitors as modulators of the activity of topoisomerase inhibitors, the effect of NU1025 on

*Received 8 May 2000*
*Revised 8 September 2000*
*Accepted 19 September 2000*

*Correspondence to:* NJ Curtin. E-mail: n.j.curtin@ncl.ac.uk

*Present address: Biomolecular Damage Group, Centre for Mechanisms of Human Toxicology, University of Leicester, UK

camptothecin- and etoposide-induced cytotoxicity, DNA strand breaks and PARP activity was investigated.

### METHODS

### Reagents

All reagents, unless stated otherwise, were obtained from Sigma (Sigma-Aldrich Company Ltd, Poole, UK) or BDH Ltd (Poole, UK). Alcohol dehydrogenase (ADH) and proteinase K were purchased from Boehringer Mannheim Biochemica (Mannheim, Germany). [2-$^{14}$C]-Thymidine (specific activity = 1.96 GBq mMol$^{-1}$) and [methyl-$^{3}$H]-thymidine (specific activity = 1.85 TBq mMol$^{-1}$) were purchased from Amersham International (Amersham, UK). Etoposide (VP16) and camptothecin were dissolved in dry DMSO to give 10 mM stock solutions, and stored at −20˚C. NU1025 was prepared as previously described (Griffin et al, 1995*b*) and dissolved in DMSO to give 100 mM stock solutions and stored at −20˚C.

### Clonogenic cell survival assay

Exponentially growing L1210 cells ($10^5$ cells ml$^{-1}$) were exposed to a range of concentrations, in duplicate, of camptothecin or etoposide in the presence or absence of 200 µM NU1025 for 16 h at 37˚C. Camptothecin preferentially kills S-phase cells and cell replication has been implicated as a determinant of cytotoxicity (Kaufmann, 1998). Therefore, since the duration of drug exposure in relation to the cell cycle time is important, cells were exposed to the drug for > one doubling time (doubling time = 12 h) to ensure that the drug was present during the S-phase of all replicating cells. All drugs were added in DMSO to give a final concentration of 1% (v/v) DMSO, and untreated controls were also exposed to 1% (v/v) DMSO. Drug exposure was stopped by centrifugation and the cells were seeded for colony formation in 0.125% (w/v) agarose (SeaKem; Flowgen Insruments Ltd, Sittingbourne, UK) in medium. Viable colonies were visualized by staining with MTT (3-[4,5-dimethylthiazol-2-yl] 2,5-diphenyltetrazolium bromide) solution in PBS (0.5 mg ml$^{-1}$) and counted. The plating efficiencies relative to control for each drug concentration/combination were calculated to give cell survival as a percentage of control. The plating efficiency for untreated controls, DMSO controls and cells treated with 200 µM NU1025 were not significantly different from 100% ($P > 0.05$ Student's unpaired *t*-test). The $LC_{50}$ and $LC_{90}$ (concentrations of drug to give 50% or 90% reduction in cell survival) were calculated using a point-to-point curve plot using Graphpad PRISM software (Graphpad Inc, San Diego, CA, USA).

### DNA strand break assay by alkaline elution

The alkaline elution technique for the quantitative analysis of DNA single-strand breakage in which fragments of DNA were separated on the basis of size using polycarbonate filters, which are neither protein- nor DNA-adsorbent was used as described by Kohn et al (1981). The alkaline elution assay has been shown previously to have a sensitivity of 1 DNA lesion/$10^7$ nucleotides. To increase the precision of the assay the samples were co-eluted with an internal standard consisting of irradiated DNA. Exponentially growing L1210 cells labelled with [2-$^{14}$C]-thymidine (14.8 KBq ml$^{-1}$) for 24 h followed by 4 h in fresh medium then treated for 16 h with the drug combinations and concentrations indicated. They were co-eluted with [methyl-$^{3}$H]-thymidine (37 KBq ml$^{-1}$)-labeled internal standard cells exposed to 3 Gy γ-radiation (using a $^{137}$Cs source, Gammacell 1000 elite; Nordion International Inc, Kanata, Canada). Single-strand break frequency, calculated as rad equivalents, was determined by comparison with a calibration curve of elution rate constant (slope determined by linear regression analysis) vs γ-radiation dose. Under the conditions used here the limit of the sensitivity of the alkaline elution assay was such that strand-break frequencies of more than 300 rad equivalents could not be measured with any degree of accuracy.

### PARP activation assay

L1210 cells were exposed to varying concentrations of camptothecin or etoposide for 6 h prior to permeabilization in hypotonic buffer and cold shock as described previously (Halldorsson et al, 1978). Briefly, cells were suspended in hypotonic buffer (9 mM HEPES, pH 7.8, 4.5% (v/v) dextran, 4.5 mM $MgCl_2$ and 5 mM DTT) at $1.5 \times 10^7$ ml$^{-1}$ on ice for 30 min, then 9 vol of isotonic buffer (40 mM HEPES, pH 7.8, 130 mM KCl, 4% (v/v) dextran, 2 mM EGTA, 2.3 mM $MgCl_2$, 225 mM sucrose and 2.5 mM DTT) was added. The reaction was started by adding 300 µl cells to 100 µl 300 µM $NAD^+$ containing [$^{32}$P]-$NAD^+$ (Amersham, UK), and terminated by the addition of 2 ml ice-cold 10% (w/v) TCA + 10% (w/v) sodium pyrophosphate. After 30 min on ice the precipitated $^{32}$P-labelled ADP-ribose polymers were filtered on Whatman GC/C filters (Whatman International Ltd, Kent, UK), washed five times with 1% (v/v) TCA, 1% (v/v) sodium pyrophosphate, dried and counted as described above.

### Apoptosis assay

A sample of 1–2 × $10^5$ control or drug-treated cells were centrifuged at 3000 rpm (600 ***g***) for 3 min and resuspended in 20 µl methanol:acetic acid (3:1 v/v) for 2 min. The cells were stained with 20 µl Hoechest 33258 (8 µg ml$^{-1}$ in PBS) and viewed using UV microscope. A minimum of 600 cells were counted and apoptotic cells, recognized by condensed chromatin, were expressed as a percentage of the total number of cells counted.

### RESULTS

### Potentiation of camptothecin and etoposide cytotoxicity by NU1025

NU1025 was not cytotoxic per se at 200 µM (% survival for a 16-h exposure was 100 ± 12%), consistent with the results from the previous study by Boulton et al (1995). Exposure to the topoisomerase I and II inhibitors camptothecin and etoposide for 16 h induced a concentration-dependent decrease in L1210 cell survival (Figure 1A and 1B). Camptothecin cytotoxicity was significantly increased by 200 µM NU1025 (*t*-test, $P \leq 0.05$), as shown in Figure 1A; pooled data from three experiments are given in Table 1. The magnitude of the potentiation was similar at low and high camptothecin concentrations, with an enhancement factor of 2.6 observed for both $LC_{50}$ and $LC_{90}$ values. In contrast there was no enhancement of etoposide cytotoxicity by NU1025 (Figure 1b, Table 1). The effects of NU1025 on etoposide cytotoxicity over a longer exposure period (24 h) were also studied, but again NU1025 had no effect (data not shown).

© 2001 Cancer Research Campaign 

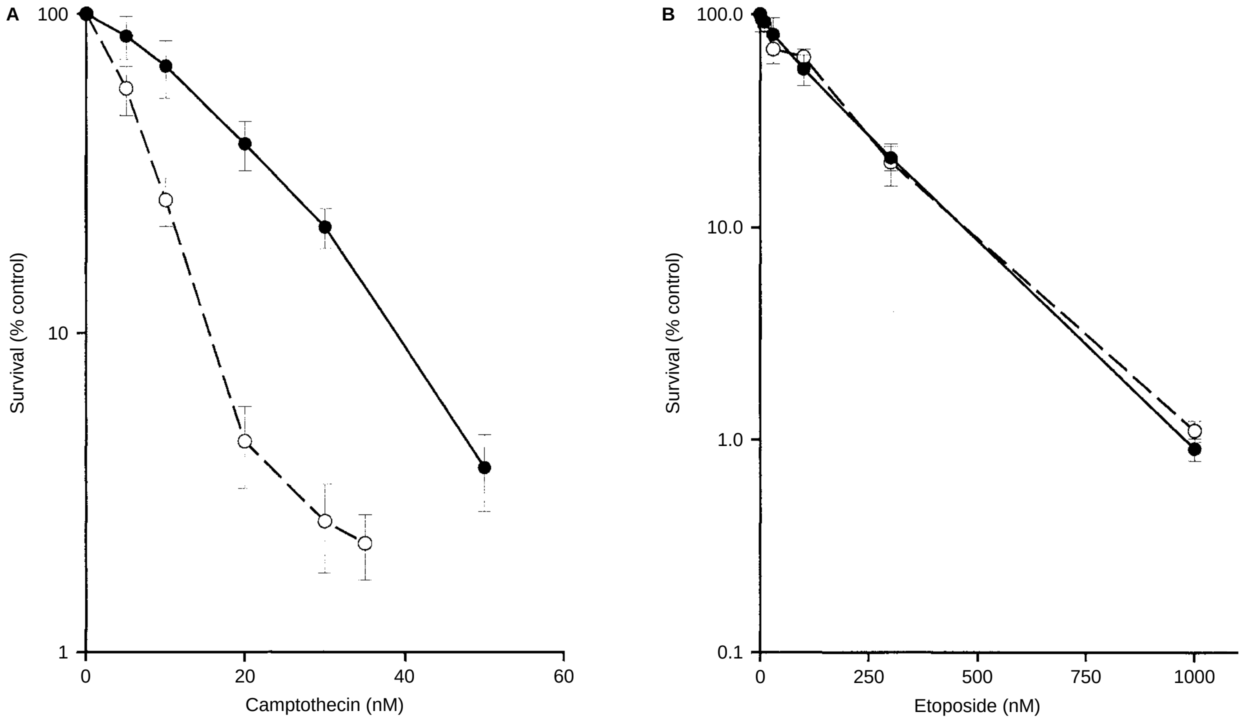

**Figure 1** Potentiation of (**A**) camptothecin and (**B**) etoposide cytotoxicity by NU1025. Cells were exposed to varying concentrations of camptothecin or etoposide in the presence (open circles) or absence (closed circles) of 200 µM NU1025 for 16 h prior to seeding for colony formation. Data (normalized to DMSO or 200 µM NU1025 alone controls) are the mean ± standard deviation of triplicate colony counts from each of the cell populations exposed in duplicate

It has been proposed by Mattern et al (1987) that reduced poly-ADP-ribosylation of topoisomerase II was responsible for the potentiation of teniposide cytotoxicity by 3AB in L1210 cells. In the studies performed by these latter authors, cells were exposed to 3-aminobenzamide prior to exposure to teniposide. To investigate if exposure to a PARP inhibitor prior to etoposide treatment resulted in increased cytotoxicity, cells were pretreated with 200 µM NU1025 for 16 h, followed by a 2, 4 or 16-h exposure to etoposide in the presence of NU1025. However, in all cases there was again no potentiation of etoposide cytotoxicity (data not shown).

**Table 1** Potentiation of camptothecin and etoposide-induced cytotoxicity by NU1025 and NU1064. Cells were exposed to camptothecin or etoposide ± 200 µM NU1025 for 16 h. The $LC_{50}$ and $LC_{90}$ values were calculated from the data shown in Figures 1A and 1B. The Enhancement factors give the relative decrease in $LC_{50}$ and $LC_{90}$ values following potentiation by NU1025. Results are expressed as the mean ± s.d. of three independent experiments.

| Cytotoxic agent | Control | + NU1025 | Enhancement factor |
|---|---|---|---|
| Camptothecin | | | |
| $LC_{50}$(nM) | 15 ± 2 | 6.1 ± 1.7[a] | 2.6 ± 0.4 |
| $LC_{90}$(nM) | 39 ± 6.5 | 15 ± 4[a] | 2.6 ± 0.3 |
| Etoposide | | | |
| $LC_{50}$(nM) | 183 ± 64 | 202 ± 62 | no potentiation |
| $LC_{90}$(nM) | 763 ± 167 | 783 ± 177 | no potentiation |

[a]Data significantly different in the presence of NU1025 (*P* = < 0.05) as shown by a paired, two-tailed, Student's *t*-test

### Effect of NU1025 on DNA strand breaks induced by camptothecin and etoposide

DNA strand breakage was measured after a 16-h exposure to the topoisomerase inhibitors in the presence or absence of 200 µM NU1025 (Figure 2). Data, expressed as rad equivalents, from three independent experiments are given in Table 2. No DNA strand breakage was detected following exposure to 200 µM NU1025 alone (Figure 2A and 2B), consistent with the observation that exposure of cells to 1 mM NU1025 for 24 h had no effect on DNA strand-break levels (Boulton et al, 1995).

**Table 2** DNA damage induced by camptothecin and etoposide ± NU1025. Cells were exposed to camptothecin or etoposide in the presence or absence of 200 µM NU1025 for 16 h prior to determination of DNA strand breakage by alkaline elution. Data are the mean ± s.d. for three independent experiments of the type shown in Figure 2

| Cytotoxic agent | DNA strand-break levels (rad equivalents) | |
|---|---|---|
| | Without NU1025 | + 200 µM NU1025 |
| Camptothecin | | |
| 0 nM | 25 ± 7 | 36 ± 8 |
| 15 nM | 33 ± 13 | 81 ± 27 |
| 40 nM | 77 ± 29 | 172 ± 25 |
| 100 nM | 261 ± 11 | 270 ± 12 |
| Etoposide | | |
| 0 nM | 43 ± 32 | 85 ± 36 |
| 150 nM | 104 ± 30 | 106 ± 39 |
| 400 nM | 198 ± 31 | 190 ± 7 |
| 800 nM | 287 ± 19 | 304 ± 17 |

 © 2001 Cancer Research Campaign

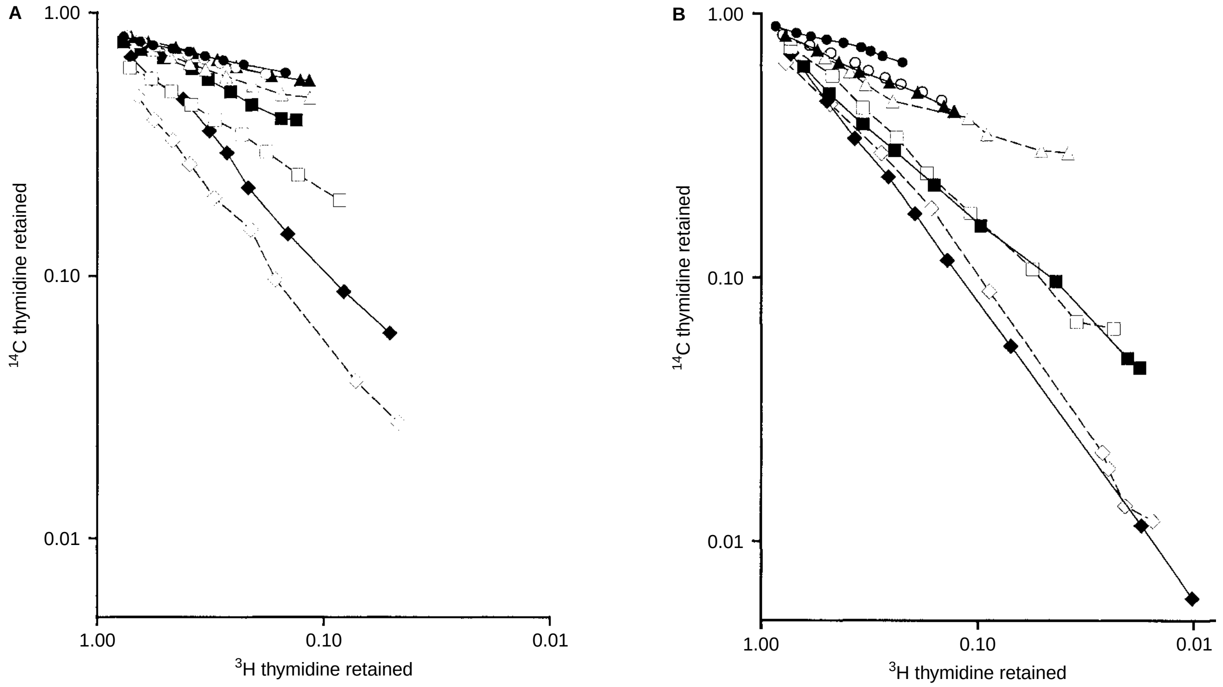

**Figure 2** Effect of NU1025 on DNA damage by (**A**) camptothecin and (**B**) etoposide. Cells were exposed to varying concentrations of camptothecin or etoposide in the presence (open symbols) or absence (closed symbols) of 200 μM NU1025 for 16 h prior to determination of DNA strand breakage by alkaline elution. Data are: control = circles, or (**A**) plus camptothecin; 15 nM = triangles; 40 nM = squares; 100 nM = diamonds and (**B**) plus etoposide; 150 nM = triangles; 400 nM = squares; 800 nM = diamonds. Representative elution profiles from duplicate samples are shown

Although exposure to an $LC_{50}$ concentration of camptothecin (15 nM) did not induce significant levels of DNA strand breaks, compared to control cells, treatment with 40 nM camptothecin (an $LC_{90}$ concentration) did produce a significant level of breaks (Figure 2A, Table 2). Consistent with the cytotoxicity data, NU1025 increased the DNA strand breakage caused by $LC_{50}$ and $LC_{90}$ concentrations of camptothecin, 2.5-fold and 2.2-fold respectively. Thus, the magnitude of the increases in camptothecin-induced DNA strand-break levels and cytotoxicity produced by NU1025 were essentially the same (2.5-fold). Maximum detectable DNA strand-break levels were produced by 100 nM camptothecin alone (i.e. 250–300 rad equivalents), and hence potentiation of DNA damage by NU1025 at this concentration could not be determined.

Treatment of cells with etoposide induced a concentration-dependent increase in DNA strand-break levels (Figure 2B and Table 2), and etoposide induced a greater number of DNA breaks than camptothecin at equitoxic concentrations. Thus, DNA strand breaks were detectable following treatment with an $LC_{50}$ concentration of etoposide (150 nM), and maximum detectable DNA strand-break levels (250–300 rad equivalents) were achieved at the $LC_{90}$ concentration of etoposide (800 nM). Consistent with the cytotoxicity data, NU1025 did not affect the DNA strand-break levels compared to those observed following treatment with etoposide alone.

### Effect of camptothecin and etoposide on whole cell PARP activity

The potential of the DNA strand breaks induced by camptothecin and etoposide to activate PARP was measured directly. Cells were exposed to varying concentrations of camptothecin or etoposide for 6 h, prior to permeabilization and analysis of PARP activity. Since the $LC_{90}$ concentration of camptothecin (40 nM) only induced a low level of strand breaks (77 rad equivalents), the higher camptothecin concentrations of 120 nM (which resulted in approximately 1% cell survival) was also used. Etoposide induced substantial DNA strand breakage at the $LC_{90}$ concentration, and hence 1 μM etoposide was used in these studies. Previous reports of PARP activation (as determined by $NAD^{+}$ depletion) following etoposide treatment have used the much higher concentrations of 17 μM (Tanizawa et al, 1989), which would be equivalent to approximately 20 × the $IC_{90}$ concentration for L1210 cells. To allow comparison with these published data, the effects of 17 μM etoposide and an equivalent supralethal concentration of camptothecin (1 μM) on PARP activity were compared. Camptothecin at both 120 nM and 1 μM caused significant activation of PARP, whereas similarly cytotoxic concentrations of etoposide (1 μM and 17 μM) had no effect on PARP activity (Figure 3).

### Induction of apoptosis by camptothecin and etoposide

It has been suggested that secondary fragmentation of DNA during apoptosis may induce PARP (Negri et al, 1993). To investigate if apoptotic DNA cleavage was responsible for the observed effects of camptothecin and etoposide on PARP activity, morphological examination of Hoechst 33258-stained cells was conducted. Apoptotic cells were identified as those with condensed chromatin, and levels of apoptotic cells were approximately 1% in control L1210 cell cultures. Exposure to 120 nM camptothecin for 16 h only induced apoptotic nuclear morphology in 8% of cells; however, 43% of cells were apoptotic following 16 h exposure to

*© 2001 Cancer Research Campaign* 

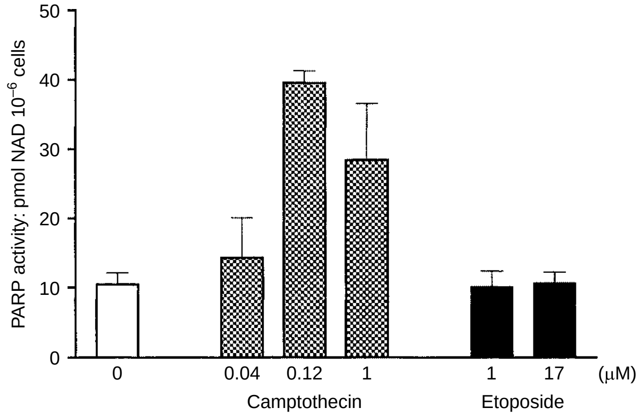

**Figure 3** PARP activity in whole cells following treatment with camptothecin and etoposide. Cells were exposed to varying concentrations of camptothecin or etoposide for 6 h prior to permeabilization and assay for PARP activity. Data are the mean ± s.d. for quadruplicate samples from each of the cell populations exposed in duplicate. 0 = control

1 μM camptothecin. Similarly, following exposure to $IC_{90}$ concentration of etoposide for 16 h only 3% of the cells were apoptotic whereas exposure to 17 μM VP16 induced apoptosis in 37% of cells.

From a comparison of the PARP activation and apoptosis data it would appear that in camptothecin-treated cells PARP activation results from the primary DNA fragmentation induced by the drug at $LC_{90}$ concentrations at early time-points. In contrast to the effects of camptothecin, primary DNA fragmentation induced by cytotoxic etoposide concentrations at early time-points does not appear to stimulate PARP activity.

### DISCUSSION

We report here an investigation of the role of PARP in topoisomerase inhibitor-mediated cytotoxicity using the novel PARP inhibitor NU1025. Using identical drug-exposure conditions NU1025 increased camptothecin-induced DNA strand breakage and cytotoxicity by a similar amount (2.2–2.6-fold), strongly suggesting that the increase in cytotoxicity was due to increased DNA strand-break levels. The activation of PARP by camptothecin-induced DNA strand breaks was investigated at concentrations at and above the $LC_{90}$, as lower concentrations produced too few DNA strand breaks to cause detectable PARP activation. Concentrations of camptothecin which resulted in ≥250 rad equivalents DNA strand breakage (120 nM, 1 μM) caused significant activation of PARP at 6 h. Since the level of apoptosis induced by 120 nM camptothecin was only 8% at 16 h, the effects of camptothecin on DNA strand breakage and PARP activation observed at this concentration were unlikely to be secondary to apoptotic DNA fragmentation. However, PARP activation following exposure to 1 μM camptothecin may be due to both camptothecin-induced and apoptosis-associated DNA breaks. Together, these data provide evidence that PARP is directly activated by camptothecin-induced DNA damage, and that inhibition of PARP increases the level of DNA strand breaks and associated cytotoxicity.

In contrast to its effect on camptothecin cytotoxicity, NU1025 had no effect on etoposide-mediated cytotoxicity or DNA strand breakage. Consistent with the lack of an effect of NU1025 on etoposide-mediated DNA strand breakage and cytotoxicity PARP activation was not observed following exposure to approximately $LC_{90}$ (1 μM) etoposide, despite the observation that significant levels of DNA strand breakage occurred. Following exposure to a supralethal (17 μM) concentration of etoposide for 16 h approximately 40% of the cells were found to be apoptotic but no PARP activation was detected at 6 h (Figure 3). Therefore, either PARP is not activated following secondary etoposide-induced apoptotic DNA fragmentation or such fragmentation occurs at a later stage. From previous studies it would appear that etoposide-induced PARP activation is dependent not only on the concentration and schedule of etoposide exposure, but also on the cell type. For example, etoposide has been found to activate PARP in HL-60, U939 and HeLa cells but not Molt 4 and CEM cells (Tanizawa et al, 1989; Kubota et al, 1990; Negri et al, 1993; Bernardi et al, 1995). Together, the failure of NU1025 to enhance etoposide cytotoxicity or DNA strand breakage, and the lack of PARP activation following etoposide treatment, indicate that PARP is not involved in etoposide cytotoxicity in L1210 cells.

The differential effect of PARP inhibition on camptothecin and etoposide cytotoxicity in L1210 cells may be due to differences in the nature of the DNA strand breaks formed by the two drugs. Etoposide induces only protein (topoisomerase II)-associated double- and single-stranded DNA breaks and cross-links. The associated proteins may prevent PARP binding to the DNA strand break, and hence PARP activation. In contrast, it has been proposed that collision between the DNA replication fork and camptothecin-topoisomerase I complex produces a protein-associated single-strand break and a non-protein-associated double-strand break 3' to the complex, (Pommier et al, 1994). Indeed, DNA double-strand ends have been detected in extracts from human colon carcinoma cells treated with camptothecin (Strumberg et al, 1999). Blunt-ended double-strand breaks are potent activators of PARP (Benjamin and Gill, 1980) and these lesions may be responsible for the activation of PARP following camptothecin treatment.

The potentiation of camptothecin by NU1025 is particularly interesting as it does not coincide with current theories of PARP involvement with BER pathways (Dantzer et al, 1999) and further work is needed to identify the lesion responsible for the activation of PARP by camptothecin. Little is known about repair of camptothecin-induced DNA damage, although an enzyme with 3'-specific tyrosyl-DNA phosphodiesterase activity has been described which may be involved in the repair of topoisomerase I-DNA complexes (Yang et al, 1996). It is conceivable that the steps subsequent to topoisomerase I removal by this repair enzyme could involve some mechanism common to the BER pathway. Recently it has been proposed that BER is accomplished by a multiprotein complex consisting of PARP, XRCC1, DNA polymerase β and DNA ligase II (Caldecott et al, 1996; Mason et al, 1998). Interestingly, EM9 cells with defective XRCC1 are hypersensitive to camptothecin (Caldecott and Jeggo, 1991) but not etoposide (Jeggo et al, 1989). BER may be associated with the repair of replication-independent camptothecin-induced DNA damage as aphidicolin, which protected wild-type cells, had only a modest protective effect in camptothecin-treated EM9 cells (Barrows et al, 1998). Similarly, PARP-deficient V79 cells are hypersensitive to topoisomerase I inhibitors (Chatterjee et al, 1990) but resistant to etoposide (Chatterjee et al, 1994). Thus it would appear that deficiencies in one component of the BER complex, as in EM9 cells, or inhibition of another component,

© 2001 Cancer Research Campaign

PARP as described in this study, sensitizes cells to camptothecin but not etoposide, implicating BER in repair of topoisomerase I-but not topoisomerase II-mediated DNA damage.

On the basis of the studies reported here, PARP inhibitors would appear to be potentially useful as resistance-modifying agents in combination with topoisomerase I inhibitor anticancer chemotherapy. Further work in this laboratory using a panel of 12 cell lines demonstrates that potentiation of topoisomerase I poisons by PARP inhibitors is not a cell line-specific phenomenon, in that in all evaluable cells NU1025 increased topotecan cytotoxicity by 1.2–5.5-fold (Delaney et al, 2000). Further investigation of the role of PARP in topoisomerase I inhibitor-mediated cytotoxicity, which may lead to a better understanding of the mechanism of topoisomerase inhibitor-mediated cytotoxicity, are warranted.

### ACKNOWLEDGEMENTS

We gratefully acknowledge the support of the Cancer Research Campaign. The NU1025 was given to us by our colleagues in the Department of Chemistry, BT Golding, RG Griffin, L Pemberton and S Srinivasan, for which we are very grateful.

### REFERENCES

Banasik M, Komura H, Shimoyama M and Ueda K (1992) Specific inhibitors of poly(ADP-ribose)synthetase and mono(ADP-ribosyl)transferase. *J Biol Chem* **267**: 1569–1575

Barrows LR, Holden JA, Anderson M and D'Arpa P (1998) The CHO XRCC1 mutant, EM9, deficient in DNA ligase III activity, exhibits hypersensitivity to camptothecin-independent of DNA replication. *Mutat Res* **408**: 103–110

Benjamin RC and Gill DM (1980) Poly(ADP-ribose) synthesis *in vitro* programmed by damaged DNA: comparison of DNA molecules containing different types of strand breaks. *J Biol Chem* **255**: 10502–10508

Bernardi R, Negri C, Donzelli M, Torti M, Prosperi E and Scovassi AI (1995) Activation of poly(ADP-ribose) polymerase in apoptotic human cells. *Biochimie* **77**: 378–384

Bernges F and Zeller WJ (1996) Combination effects of poly(ADP-ribose) polymerase inhibitors and DNA-damaging agents in ovarian tumour cell lines – with special reference to cisplatin. *J Cancer Res Clin Oncol* **122**: 665–670

Boulton S, Pemberton LC, Porteous JK, Curtin NJ, Griffin RJ, Golding BT and Durkacz BW (1995) Potentiation of temozolomide-induced cytotoxicity: a comparative study of the biological effects of pol(ADP-ribose)polymerase inhibitors. *Br J Cancer* **72**: 849–856

Bowman KJ, Curtin NJ, Golding BT, Griffin RJ and White A (1998) Potentiation of anticancer agent cytotoxicity by the potent poly(ADP-ribose) polymerase inhibitors, NU1025 and NU1064. *Br J Cancer* **78**: 1269–1277

Caldecott K and Jeggo P (1991) Cross-sensitivity of γ-ray-sensitive hamster mutants to cross-linking agents. *Mutat Res DNA Repair* **255**: 111–121

Caldecott KW, Aofouchi S, Johnson P. and Shall S (1996) XRCC1 polypeptide interacts with DNA polymerase beta and possibly poly(ADP-ribose) polymerase, and DNA ligaseIII is a novel molecular nick-sensor in vitro. *Nucleic Acids Res* **24**: 4387–4394

Chatterjee S, Cheng M-F, Trivedi D, Petzold SJ and Berger NA (1990) Camptothecin hypersensitivity in poly(adenine diphosphate-ribose) polymerase-deficient cell lines. *Cancer Commun* **1**: 401–407

Chatterjee S, Cheng M-F, Berger SJ and Berger NA (1994) Induction of $M_r$ 78,00 glucose-related stress protein in poly(adenosine diphosphate-ribose) polymerase-and nicotinamide adenine dinucleotide-deficient V70 cell lines and its relation to resistance to the topoisomerase II inhibitor etoposide. *Cancer Res* **54**: 4405–4411

Dantzer F, Schreiber V, Niedergang C, Trucco C, Flatter E, de la Rubia G, Oliver J, Rolli V, Menissier-de Murcia J and de Murcia G (1999) Involvement of poly(ADP-ribose) polymerase in base excision repair. *Biochimie* **81**: 69–75

Darby MK, Schmitt B, Jongstra-Bilen J and Vosberg HP (1985) Inhibition of calf thymus type II topoisomerase by poly (ADP-ribosylation). *EMBO J* **4**: 2129–2134

Daugherty JP, Simpson TA Jr. and Mullins DW Jr. (1988) Effect of hyperthermia and doxorubicin on nucleoid sedimentation and poly(ADP-ribose)polymerase activity in L1210 cells. *Cancer Chemother Pharmacol* **21**: 229–232

Delaney CA, Wang L-Z, Kyle S, Srinivasan S, White AW, Calvert AH, Curtin NJ, Durkacz BW, Hostomsky Z, Maegley K, Golding BT, Griffin RG and Newell DR (2000) Potentiation of temozolomide and topotecan growth inhibition and cytotoxicity by novel poly(ADP-ribose) polymerase inhibitors in a panel of human tumour cell lines. *Clin Cancer Res* **6**: 2860–2867

de Murcia G and Menissier-de Murcia J (1994) Poly(ADP-ribose)polymerase: a molecular nick-sensor. *TIBS* **19**: 172–176

Eriksson C, Busk L and Brittebo EB (1996) 3-Aminobenzamide: effects on cytochrome P450-dependent metabolism of chemicals and on the toxicity of dichlobenil in the olfactory mucosa. *Toxicol Appl Pharmacol* **136**: 324

Ferro AM and Olivera BM (1984) Poly (ADP-ribosylation) of DNA topoisomerase I from calf thymus, *J Biol Chem* **259**: 547–554

Griffin RJ, Curtin NJ, Newell DR, Golding BT, Durkacz BW, and Calvert AH (1995*a*). The role of inhibitors of poly(ADP-ribose)polymerase as resistance modifying agents in cancer therapy. *Biochimie* **77**: 364–367

Griffin RJ, Pemberton LC, Rhodes D, Bleasdale C, Bowman K, Calvert AH, Curtin NJ, Durkacz BW, Newell DR, Porteous JK and Golding BT (1995*b*). Novel potent inhibitors of the DNA repair enzyme poly(ADP-ribose)polymerase (PARP). *Anti-Cancer Drug Design* **10**: 507–514

Griffin RJ, Srinivasan S, White AW, Bowman K, Calvert AH, Curtin NJ, Newell DR and Golding BT (1996) Novel benzimidazole and quinazolinone inhibitors of the DNA repair enzyme, poly (ADP-ribose)polymerase. *Pharmaceut Sci* **2**: 43–47

Halldorsson H, Gray DA and Shall S (1978) Poly(ADP-ribose)polymerase activity in nucleotide permeable cells. *FEBS Lett* **85**: 349–352

Jeggo PA, Caldecott K, Pidsley S and Banks GR (1989) Sensitivity of Chinese hamster ovary mutants defective in DNA double strand break repair to topoisomerase II inhibitors. *Cancer Res* **49**: 7057–7063

Kaufmann SH (1998) Cell death induced by topoisomerase-targeted drugs: more questions than answers. *Biochim Biophys Acta* **1400**: 185–211

Kaufmann SH, Charron M, Burke PJ and Karp JE (1995) Changes in topoisomerase I levels and localisation during myeloid maturation *in vitro* and *in vivo*. *Cancer Res* **55**: 1255–1260

Kohn KW, Ewig RA, Erickson LC and Zwelling LA (1981) Measurement of strand breaks and cross-links by alkaline elution. In *DNA repair: a laboratory manual of research procedures*. Friberg EC and Hanawalt PC (eds) pp 379–401. Marcel Dekker Inc: New York

Kubota M, Tanizawa A, Hashimoto H, Shimizu T, Takimoto T, Kitoh T, Akiyama Y and Mikawa H (1990) Cell type dependent activation of poly(ADP-ribose) synthesis following treatment with etoposide. *Leukaemia Res* **14**: 371–375

Marks DI and Fox RM (1991) DNA damage, poly(ADP-ribosyl)ation and apoptotic cell death as a potential common pathway of cytotoxic drug action. *Biochem Pharmacol* **42**: 1859–1867

Mason M, Niedergang C, Schreiber V, Muller S, Menissier deMurcia J and deMurcia G (1998) XRCC1 is specifically associated with poly(ADP-ribose) polymerase and negatively regulates its activity following DNA damage. *Mol Cell Biol* **18**: 3563

Mattern MR, Mong S-M, Bartus HF, Mirabelli CK, Crooke ST, and Johnson RK (1987) Relationship between the intracellular effects of camptothecin and the inhibition of DNA topoisomerase I in cultured L1210 cells. *Cancer Res* **47**: 1793–1798

Milam KM, Thomas GH and Cleaver JE (1986) Disturbances in DNA precursor metabolism associated with exposure to an inhibitor of poly(ADP-ribose) synthetase. *Exp Cell Res* **165**: 260

Moses K, Harris AL and Durkacz BW (1988*a*) Adenosine-diphosphoribosyl transferase inhibitors can protect against or potentiate the cytotoxicity of S-phase acting drugs. *Biochem Pharmacol* **37**: 2155

Moses K, Harris AL and Durkacz BW (1988*b*) Synergistic enhancement of 6-thioguanine by ADP-ribosyltransferase inhibitors. *Cancer Res* **48**: 5650

Moses K, Willmore E, Harris AL and Durkacz BW (1990) Correlation of enhanced 6-mercaptopurine cytotoxicity with increased phosphoribosylpyrophosphate levels in Chinese hamster ovary cells treated with 3-aminobenzamide. *Cancer Res* **50**: 1992

Negri C, Bernardi R, Braghetti A, Astaldi Ricotti GCB and Scovassi IA (1993) The effect of the chemotherapeutic drug VP-16 on poly(ADP-ribosylation) in apoptotic HeLa cells. *Carcinogenesis* **14**: 2559–2564

Pommier YM, Leteurtre F, Fesen MR, Fujimori A, Bertrand R, Solary E, Kohlhagen G and Kohn KW (1994) Cellular determinants of sensitivity and resistance to DNA topoisomerase inhibitors *Cancer Invest* **12**: 530–542

Strumberg D, Pilon AA, Smith M, Malkas LH and Pommier Y (1999) Selective inhibition of lagging strand DNA replication by

© 2001 Cancer Research Campaign 

camptothecin-induced topoisomerase I-poisoning. *Proc Am Assoc Cancer Res* **40**: 208

Suto MJ, Turner WR, Arundel-Suto CM, Werbel LM, and Sebolt-Leopold JS (1991) Dihydroisoquinolinones: the design and synthesis of a new series of potent inhibitors of poly(ADP-ribose)polymerase. *Anti-Cancer Drug Design* **7**: 107–117

Tanizawa A, Kubota M, Hashimoto H, Shimizu T, Takimoto T, Kitoh T, Akiyama Y and Mikawa H (1989) VP-16-induced nucleotide pool changes and poly (ADP-ribose) synthesis: the role of VP-16 in interphase death. *Exp Cell Res* **185**: 237–246

Yang S-W, Burgin AB, Huizenga BN, Robertson CA, Yao KC and Nash HA (1996) A eukaryotic enzyme that can disjoin dead-end covalent complexes between DNA and type 1 topoisomerases. *Proc Natl Acad Sci USA* **93**: 11534–11539

Zwelling LA, Kerrigan D, Pommier Y, Michaels S, Steren A and Kohn KW (1982) Formation and resealing of intercalator-induced DNA strand breaks in permeabilised L1210 cells without the stimulated synthesis of poly (ADP-ribose) *J Biol Chem* **257**: 8957–8963

 *© 2001 Cancer Research Campaign*
